# Fetal Programming of the Endocrine Pancreas: Impact of a Maternal Low-Protein Diet on Gene Expression in the Perinatal Rat Pancreas

**DOI:** 10.3390/ijms231911057

**Published:** 2022-09-21

**Authors:** Louise Winkel, Morten Rasmussen, Louise Larsen, Louise T. Dalgaard, Jens H. Nielsen

**Affiliations:** 1 Department of Biomedical Sciences, University of Copenhagen, 2200 Copenhagen, Denmark; 2 Department of Science and Environment, Roskilde University, 4000 Roskilde, Denmark

**Keywords:** pancreas, beta cell, perinatal gene expression, fetal metabolic programming, lipid metabolism, sterol metabolic process, beta cell maturation, neurogenin *3 (Neurog3*), hepatocyte nuclear factor (*Hnf)-1α*, sterol response element binding factor (*Srebf)-1*, Srebf2, alpha fetoprotein (*Afp*), angiopoietin-like (*Angptl)4*, growth arrest specific (*Gas6*), dual-specificity phosphatase (*Dusp*)*6*, legumain (*Lgmn*), ETS variant (*Etv*)*5*, anterior gradient (*Agr*)*2*, placenta-specific (*Plac*)*8*

## Abstract

In rats, the time of birth is characterized by a transient rise in beta cell replication, as well as beta cell neogenesis and the functional maturation of the endocrine pancreas. However, the knowledge of the gene expression during this period of beta cell expansion is incomplete. The aim was to characterize the perinatal rat pancreas transcriptome and to identify regulatory pathways differentially regulated at the whole organ level in the offspring of mothers fed a regular control diet (CO) and of mothers fed a low-protein diet (LP). We performed mRNA expression profiling via the microarray analysis of total rat pancreas samples at embryonic day (E) 20 and postnatal days (P) 0 and 2. In the CO group, pancreas metabolic pathways related to sterol and lipid metabolism were highly enriched, whereas the LP diet induced changes in transcripts involved in RNA transcription and gene regulation, as well as cell migration and apoptosis. Moreover, a number of individual transcripts were markedly upregulated at P0 in the CO pancreas: growth arrest specific 6 (*Gas6*), legumain (*Lgmn*), Ets variant gene 5 (*Etv5*), alpha-fetoprotein (*Afp*), dual-specificity phosphatase 6 (*Dusp6*), and angiopoietin-like 4 (*Angptl4*). The LP diet induced the downregulation of a large number of transcripts, including neurogenin 3 (*Neurog3*), *Etv5*, *Gas6*, *Dusp6*, signaling transducer and activator of transcription 3 (*Stat3*), growth hormone receptor (*Ghr*), prolactin receptor (*Prlr*), and Gas6 receptor (AXL receptor tyrosine kinase; *Axl*), whereas upregulated transcripts were related to inflammatory responses and cell motility. We identified differentially regulated genes and transcriptional networks in the perinatal pancreas. These data revealed marked adaptations of exocrine and endocrine in the pancreas to the low-protein diet, and the data can contribute to identifying novel regulators of beta cell mass expansion and functional maturation and may provide a valuable tool in the generation of fully functional beta cells from stem cells to be used in replacement therapy.

## 1. Introduction

Type 2 diabetes (T2D) is characterized by an imbalance between the insulin demand and insulin supply by the pancreatic beta cells. Previously, peripheral insulin resistance was considered to be the main cause of T2D, but now it is recognized that an impairment of the beta cell function is a major determinant in the pathogenesis of T2D. Studies of risk genes in both the rare monogenic forms of T2D, maturity onset diabetes of the young (MODY), and the common obesity-related T2D have shown that most of the risk alleles are expressed in pancreatic beta cells [1]. In addition to genetic risk factors, accumulating evidence during the last two decades has pointed towards the importance of the intrauterine environment in the later development of diseases, including metabolic disorders such as T2D [2]. The concepts of “fetal programming” and the “developmental origin of health and disease” have become widely accepted. Thus, maternal malnutrition with both too few and too many calories increases the risk of developing obesity and T2D [1]. Several organs including the endocrine pancreas are sensitive to metabolic programming by the fetal and neonatal environment [1]. A protein-deficient diet during gestation is an established model for malnutrition in rats that results in a low birth weight and reduced beta cell mass relative to the body weight in the offspring, which will develop into T2D later in life [3].

Major advances have been achieved in unraveling the molecular regulation of the formation of beta cells in the rodent embryonic pancreas [4,5,6]. In addition, considerable progress has been made in describing the growth dynamics of fetal, neonatal, and adult endocrine cells [7,8,9]. However, only a few studies have focused on the mechanisms underlying the burst of beta cell formation and subsequent functional maturation in the perinatal period [10,11,12]. In this time period, from embryonic day 20 (E20) to postnatal day 2 (P2), the insulin content of the pancreas increases by ten-fold and the insulin secretion by six-fold [10,13]. Understanding the molecular mechanisms that regulate the generation of beta cells in the perinatal period may, thus, be of great value in the design of cell-based therapies and in regenerative medicine.

The aim of the present study was to investigate the expression patterns and transcriptional networks of pancreatic endocrine genes during the period of perinatal beta cell expansion and maturation. Since a major part of this beta cell expansion occurs via neogenesis from progenitors that may be located outside the endocrine compartment [11,14], and since signals from non-endocrine cells may play a critical role in this process, microarray analyses of the perinatal pancreas were performed on samples from the total pancreas. We analyzed pancreas samples from both the offspring of dams fed a normal control (CO) diet and the offspring of dams fed a low-protein (LP) diet during gestation. By investigating transcripts regulated during the perinatal period in both CO pups and LP pups, it may be possible to identify genes that are involved in the regulation of beta cell neogenesis, expansion, and maturation and to qualify key sites for upregulating the functional beta cell mass in vivo as well as ex vivo.

## 2. Results

### 2.1. Gene Regulation in Normal Perinatal Rat Pancreas from E20 to P2

**Figure 1:**
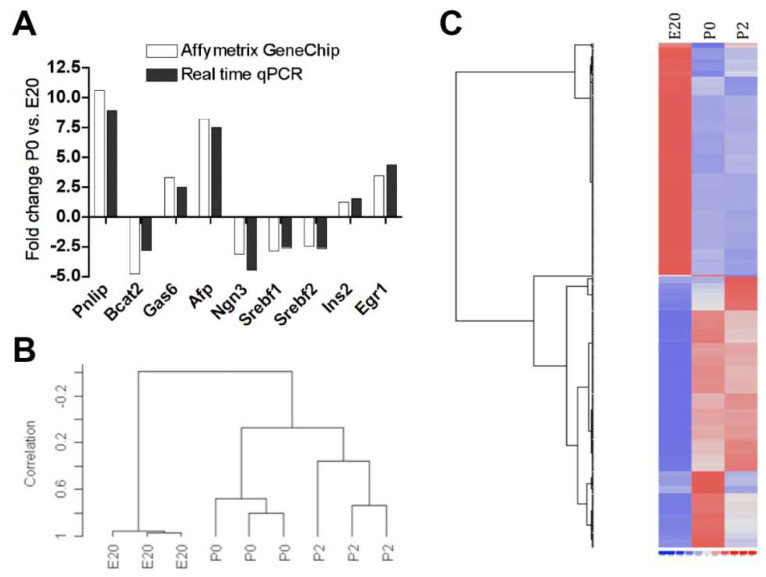
(**A**) Comparison of fold changes (FCs) in mRNA expression levels between P0 and E20 as obtained by hybridization to Affymetrix GeneChip versus RTqPCR for the number of selected up- and downregulated genes. There are no error bars, since data are presented as fold changes. Note: Pnlip: pancreatic lipase; Bcat2: branched-chain amino acid transaminase 2; Gas6: growth-arrest-specific 6; Afp: alpha-fetoprotein; Ngn3: neurog3; Srebf1/2: sterol regulatory element binding protein 1/2; Ins2: insulin 2; Pdx1: pancreatic duodenal homeobox 1; Egr1: early growth response factor 1. (**B**) Dendrogram for clustering using centered correlation and average linkage. Samples from P0 and P2 cluster closer together, indicating a more similar profile compared to samples from E20. (**C**) Heat map generated from the dCHIP hierarchical cluster analysis. Note: E20: embryonic day 20; P0: day 0 postpartum; P2: day two postpartum. Blue: Low expression. Red: High expression.

A total of 1004 (723 annotated) non-redundant genes were differentially regulated in rat pancreas samples between E20 and birth (P0 vs. E20), while 305 (200 annotated) were differentially regulated between birth and 2 days post partum (P2 vs. P0), of which 167 intersected. Of these 1004 genes, 524 were >1.5-fold up- or downregulated at P0 vs. E20 and 54 at P2 vs. P0 (Supplementary Table S1), of which 45 intersected. Thus, the most dramatic change in gene expression both in respect to the number of regulated genes and degree of regulation was observed between E20 and birth. In support of this, the cluster analysis and heat map show closer relations between P2 and P0 than to E20 (Figure 1). Table 1 lists the most up- and downregulated transcripts. To verify the array hybridizations, a reverse transcription quantitative (RT-q)PCR was performed on selected up- and downregulated genes and the degrees of regulation were compared. The RTqPCR showed a high degree of concordance to the array signals (Figure 1A).

### 2.2. Localization of Alpha-Feto Protein (Afp) in Perinatal Rat Pancreas

**Figure 2:**
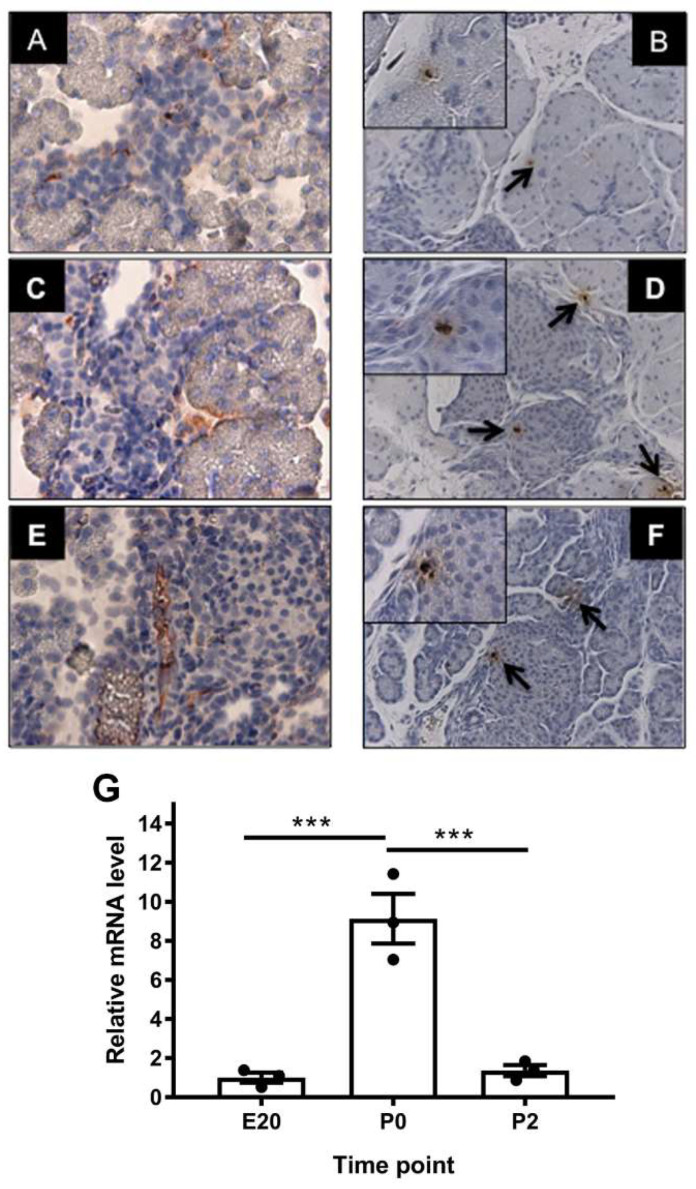
Expression and cellular localization of *Afp* in rat pancreas samples at E20, P0, and P2. (**A**,**C**,**E**) In situ hybridization of *Afp* mRNA using a dioxigenin-labeled LNA™ probe targeted against the same rat *Afp* sequence as the primers for RTqPCR (Qiagen, Exiqon, Vedbaek, Denmark) at E20 (**A**), P0 (**C**), and P2 (**E**). *Afp* is primarily expressed in association with islets of Langerhans in vessels and intra-islet capillaries. (**B**,**D**,**F**) Immunohistochemical detection (200× magnification) of Afp protein in 3 µm tissue sections from rat pancreas samples at E20 (**B**), P0 (**D**), and P2 (**F**). Afp is primarily detected in association with the endocrine compartments or ducts (arrows). Inserts at 400× magnification. (**G**) RTqPCR of *Afp* mRNA in rat pancreas samples at E20, P0, and P2. The mRNA values are normalized to *Rpl13alpha*. *Afp* mRNA expression is significantly increased at P0 compared to E20 and P2 (two-tailed *t*-test, *p* < 0.01). Note: *** *p* < 0.001.

As *Afp* is liver-restricted in adults and was found to be expressed in considerable amounts in the perinatal pancreas, we evaluated the expression and localization of *Afp* during the perinatal period. Significant *Afp* expression was also consistently observed in low-scatter non-granulated cells (considered endocrine progenitors) of isolated neonatal (P2–P3) rat islets, whereas it was expressed at almost undetectable levels in adult beta cells (published in E-GEOD-47174) [15]. We investigated the expression pattern and cellular origin of this protein at E20, P0, and P2 and found localization of *Afp* mRNA and protein associated with the endocrine compartment within or in close proximity to vessels and intra islet capillaries (Figure 2), but not in differentiated endocrine cells.

### 2.3. Clustering and Functional Annotation of Profiles

**Figure 3:**
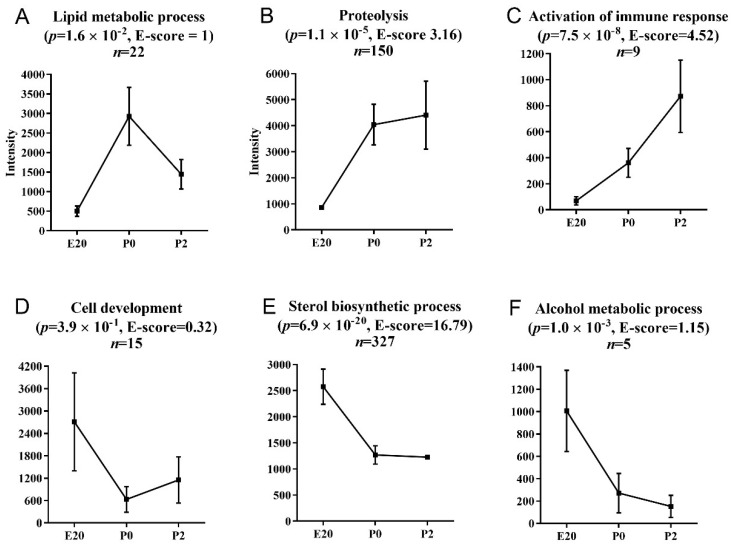
Functionally annotated gene clusters in normal perinatal rat pancreas samples. Differentially regulated transcripts in perinatal rat pancreas were clustered according to expression profiles over time and analyzed for enriched functional annotations using DAVID Functional Annotation Clustering. The most significantly enriched biological process in the highest scoring cluster is presented above each graph. Data points represent mean intensities of the associated gene cluster at the given time point. (**A**) Lipid metabolic process, (**B**) proteolysis, (**C**) activation of the immune system, (**D**) cell development, (**E**) sterol biosynthetic process, (**F**) alcohol metabolic process. Error bars (SE) are included to illustrate the variability in expression levels. *p*-value: modified one-tailed Fisher’s exact probability value (EASE score); E-score: enrichment score, the geometric mean (in -log scale) of a member’s *p*-values (EASE scores) in the corresponding annotation cluster.

The regulated transcripts can be clustered into 6 expression profile clusters (Figure 3). Profiles A, D, and F are not significantly enriched in transcripts belonging to particular functional categories (low E-scores). Profile A consists of transcripts transiently upregulated at P0 and potentially correlated with the recognized expansion of the functional beta cell mass in this time period. A number of the transcripts in this cluster encode transcription factors known to control lipid metabolic processes such as nuclear receptor subfamily 1 group D member 2 (*Nr1d2*), nuclear receptor subfamily 4 group A member 1 (*Nr4a1*), and immediate early response 3 (*Ier3*). The regulated transcripts also include Afp, dual-specificity phosphatase 6 (*Dusp6*), and angiopoietin-like 4 (*Angptl4*). These transcripts encode proteins involved in the development and regulation of cellular proliferation and differentiation [16,17], as well as glucose homeostasis and insulin sensitivity [17]. *Angptl4* is a homologue of the previously identified betatrophin (*Angptl8*) [18]. 

**Figure 4:**
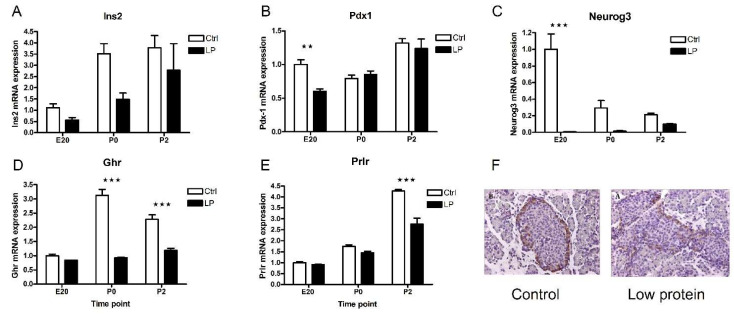
Relative *Ins2, Pdx-1, Neurog3, Ghr,* and *Prlr* mRNA expression in perinatal rat pancreas from control (Ctrl) and low-protein (LP) diet animals. Messenger RNA levels measured using RTqPCR in whole rat pancreas samples at days E20, P0, and P2. Values are normalized to levels at E20 within each data set, *n* = 3–6. Data are given as means + SEM. (**A**) *Ins2*, (**B**) *Pdx1*, (**C**) *Neurog3*, (**D**) *Ghr*, (**E**) *Prlr*, and (**F**) immunohistochemical stainings for Neurog3 in the perinatal rat pancreas at P0 (200× magnification). Data in panels (**A**–**E**) were tested using 2-way ANOVA with Bonferroni post tests for control vs. LP animals. Asterisks indicate significance levels of individual comparisons. Note: ** *p* < 0.01, *** *p* < 0.001.

The two largest clusters consist of transcripts are either upregulated (Figure 3B) or downregulated (Figure 3E) >1.5-fold at P0 compared to E20 but remain unchanged at P2 vs. P0. Terms belonging to the Gene Ontology category Proteolysis are over-represented in the profile with upregulated transcripts at P0 (B) and include *pancreatic lipase* (*Pnlip*), *chymotrypsin* (*Ctrc*), and *preprotrypsinogen IV* (*Try4*), which indicates perinatal maturation of the exocrine function. However, the cluster also contains genes characteristic of endocrine non-beta cells, such as *pancreatic polypeptide* (*Ppy*), *somatostatin* (*Sst*). and *glucagon* (*Gcg*). Profile B furthermore includes *insulin 2* (*Ins2*) (Figure 4A), *growth hormone receptor* (*Ghr*) (Figure 4D), *thyrotropin releasing hormone* (*Trh*), *paired box gene 6* (*Pax6*), and members of the *regenerating islet-derived* (*Reg*) gene family (*Reg1a, Reg3a, Reg3b, Reg3g*). Ghr is expressed in beta cells and mediates their proliferation [19]. Trh is expressed in beta cells and stored in the insulin granules. The expression coincides with the perinatal maturation, but is lower in fetal than in adult islets. The secretion is stimulated by glucose, and Trh has the potential to prevent apoptosis and promote beta cell proliferation [20,21].

In addition, transcripts not normally or only recently associated with pancreatic development and function are upregulated at birth, such as *growth-arrest-specific 6* (*Gas6*), *legumain* (*Lgmn*), and *Ets variant gene 5* (*Etv5*), which are recently recognized regulators of tissue regeneration or remodeling in extrapancreatic sites such as the liver and testes [22,23,24,25,26,27,28,29]. *Gas6* was expressed in beta cells but was reduced in LP offspring, suggesting the premature maturation of the beta cells [29]. In addition, the trefoil factor *TFF1* showed upregulation in the LP group at P0 and P2 (Table 2). The trefoil factors *TFF1*, *TFF2*, and *TFF3* are small peptides that can influence cell migration and adhesion. *TFF3* expression was increased in the perinatal pancreas of LP rats at P0 and decreased at P2 (Supplementary Table S1). The treatment of the islets with human growth hormone (hGH) stimulates *TFF3* expression and the spreading of the cells, suggesting that *TFF3* may promote the maturation of the newly formed beta cells [30,31]. A recent review has summarized the pathological and therapeutic roles of *TFF3* [32].

The profile containing downregulated genes at birth (Figure 3E) is highly enriched in transcripts involved in sterol biosynthesis. These include *farnesyl-diphosphate farnesyltransferase 1* (*Fdft1*) and *3-hydroxy-3-methylglutaryl-coenzyme A synthase 1 (soluble)* (*Hmgcs1*), as well as *insulin-induced gene 1* (*Insig1*), *squalene epoxidase* (*Sqle*), and *Srebf1/Srebf2*. Srebf1 and Srebf2 are members of the membrane-bound transcription factor basic helix–loop–helix (bHLH) leucine zipper family, and both control multiple pathways required for the regulation of beta cell function [33,34].

The small cluster of transcripts with progressively increasing expression levels over time (Figure 3C) is enriched in genes associated with the activation of the immune response, including *complement component 4B* (*C4b*), *complement factor B* (*Cfb*), and *complement factor D* (*Cfd*, also known as adipsin). The last small cluster of transcripts decreasing over time to very low levels at P2 is enriched in ‘alcohol metabolic processes’ with *aldolase B* (*Aldob*), *carbonic anhydrase XII* (*Car12*), *diacylglycerol O-acyltransferase 2* (*Dgat2*), *Egl-9 family hypoxia-inducible factor 31* (*Egln3*), and *histidine carboxylase* (*Hdc*), most of which can be related to the metabolic substrate switch following birth (*Aldob*, *Dgat2*, *Egln3*, *Hdc*).

### 2.4. Pathway Analysis of Perinatally Regulated Transcripts

**Figure 5:**
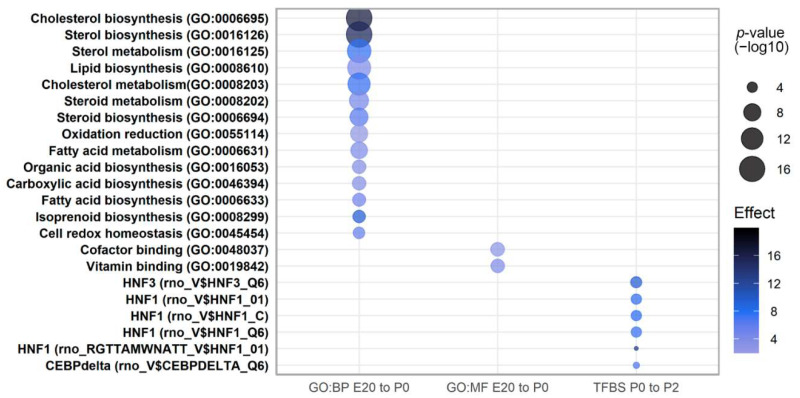
Gene Ontology biological processes and molecular functions,) which are significantly over-represented in the gene lists of differentially expressed genes at E20 vs. P0, and significantly enriched transcription factor binding sites (TFBS) based on differentially regulated genes between P0 and P2.

A pathway analysis was performed on differentially regulated transcripts at P0 vs. E20 and P2 vs. P0 using Gene Ontology terms and enrichment for transcription factor binding sites (TFBS). Molecular function terms were enriched for ‘co-factor binding’, ‘vitamin binding’, ‘co-enzyme binding’, and oxido-reductase activities (Figure 5), whereas biological process terms were highly enriched for various parts of cholesterol, sterol, steroid, and fatty acid processes (Figure 5) for transcripts regulated from E20 to P0. These enriched terms stem in part from the downregulated transcripts belonging to clusters E and F (Figure 3). There were no significant enrichments for TFBS for E20 vs. P0; however, for the comparison of P0 vs. P2, binding sites for the transcription factor hepatocyte nuclear factor 1 alpha (Hnf-1α) are more frequent among regulated transcripts. Four different binding matrices for Hnf-1α are significantly enriched (Figure 5). Hnf-1α is very important for beta cell formation and adult beta cell function, and HNF-1α mutations are the most common cause of MODY subtype 3. Furthermore, binding sites for C/Ebp-δ and Hnf3 (Foxa2) are also significantly enriched in promoters of transcripts regulated from P0 to P2.

Thus, the pathway analysis suggests that from E20 to P0, the most enriched and important regulated pathways are involved in sterol and lipid metabolic processes, whereas in the period from P0 to P2, beta cell formation becomes more critical, as illustrated by the enrichment for the binding sites for the beta-cell-specific Hnf1 following birth.

### 2.5. Characterization of the Low-Protein Malnutrition Model

To further characterize the perinatal pancreas gene expression network regarding beta cell relevant transcripts, we employed a fetal malnutrition protocol known to induce delayed beta cell development and maturation. Rat pups from dams fed a low-protein (LP) diet were significantly smaller than control (CO) pups (LP: 5.41 g ± 0.07 g vs. CO 6.27 g ± 0.11 g, mean ± SEM, *p* < 0.001). This treatment model has previously been shown to cause impaired beta cell development and glucose intolerance in adult animals [35]. We characterized the model using RTqPCR to assess transcripts that are essential for beta cell development, proliferation, and function (*Ins2*, *Neurog3*, *Pdx1*, *Ghr,* and *Prlr*). A common feature of these transcripts is the downregulation by the low-protein diet. Where the insulin expression rose at P0 in the CO pancreas, the LP pancreas showed a delayed rise in mRNA expression (Figure 4A), and this pattern was similar for the *Pdx1* mRNA (Figure 4B). The *Ghr* mRNA levels were markedly suppressed by the LP diet, while the *Prlr* mRNA levels were decreased postnatally (P2) (Figure 4D,E). *Neurog3* was suppressed significantly by the LP diet, especially at E20, while there was a gradual increase in the LP pancreas over time to P2, whereas the control pancreas showed decreasing levels of *Neurog3* (Figure 4C). This was suggestive of a delayed and impaired differentiation of beta cells in the LP animals. The immunohistochemical staining for *Neurog3* confirmed the lower expression at P0 (Figure 4F). Thus, a number of markers of beta cell differentiation, neogenesis, and proliferation were negatively affected by the LP model.

### 2.6. Transcripts Regulated by Maternal Low-Protein Diet

**Figure 6:**
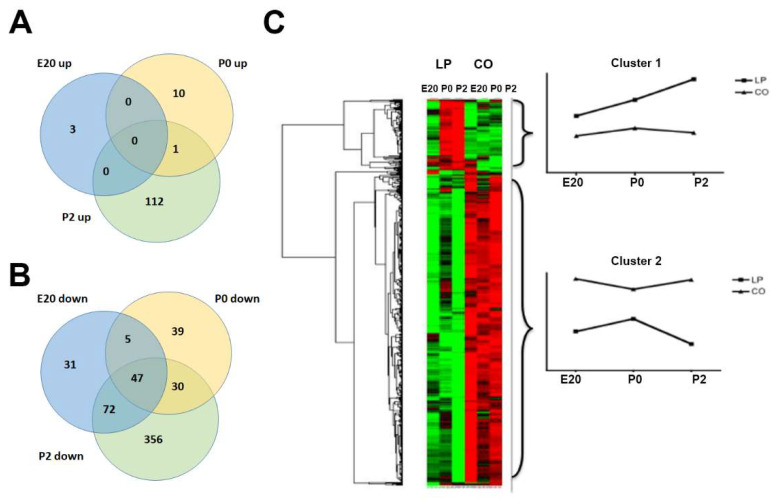
Cluster analysis of genes regulated by gestational low-protein diet. (**A**,**B**) Venn diagrams based on lists of upregulated (**A**) and downregulated (**B**) genes in the perinatal pancreas (E20, P0, and P2) programmed by the low-protein diet during gestation. (**C**) Heat map and cluster analyses of low-protein diet-regulated genes in the perinatal period. LP: Low-protein; CO: control. Green: Upregulated and Red: Down regulated.

In order to identify gene expression networks involved in the impaired function of beta cells in the LP model, microarray analyses were carried out. Here, 705 genes were differentially regulated in pancreas samples between LP and CO groups, the majority of which (578) were downregulated and 127 of which were upregulated in the LP group. The top regulated transcripts by maternal LP diet are listed in Table 2. Clustering was performed to assess the impact of the LP diet on gene expression at different time points (Figure 6). The test results show that the two dietary groups at E20 are branched together as a separate unity apart from P0 and P2 samples, whereas LP diet samples already at P0 cluster separately from the CO samples. Thus, at birth, the gene expression patterns clearly distinguish LP from CO diet samples. The heat map generated using unsupervised hierarchical clustering based on the micro-array data (Figure 6) shows two major cluster profiles. Cluster 1 includes transcripts upregulated in LP animals with a general increase over time from E20 to P2 compared to the CO group. Cluster 2 contains genes that are on average less expressed across the three time points in LP animals, with a further small decrease at P2. These include *Neurog3*, *Etv5*, *Stat3*, notch receptor 1 (*Notch1*), *SP1*, *Gas6*, and *Prlr,* as well as SWI/SNF-related, matrix-associated, actin-dependent regulator of chromatin, subfamily A, member 4 (*Smarca 4*). The prolactin receptor (Prlr) is required for the adaptation of the increased beta cell mass in pregnancy [19]. Neurog 3 (Ngn3) plays an essential role in beta cell differentiation and the expression seems to be coordinated in the maternal and fetal pancreas by circulating factors during pregnancy [36].

Cluster 1 (upregulated in LP vs. CO) consists of genes related to the inflammatory response such as chemokine (C–C motif) ligand 11 (*Ccl11*), caspase 4 (*Casp4*), complement component 5 (*C5*), and PYD and CARD-domain-containing (*Pycard*). This could indicate an increased state of inflammation in the LP group. Interestingly, in animal models, intrauterine growth restriction (IUGR) has been shown to be associated with activation of the immune system, including increased levels of inflammatory cytokines such as interlukin-1b that may contribute to the development of beta cell dysfunction and T2D [37]. The upregulation of bone morphogenetic protein 2 (*Bmp2*) in the LP group is compatible with reduced beta cell proliferation [38]. Furthermore, changes at P2 were more distinct than at E20 and P0. At P2, 617 genes significantly changed between LP and CO, while 158 and 132 genes differed on days E20 and P0, respectively. The most upregulated transcript *was anterior gradient 2* (*Agr2*), upregulated 44-fold at P2, which encodes a protein disulfide isomerase that is important for the production of mucin, but which is also a prometastatic factor for pancreatic cancer [39,40]. Placenta-specific 8 (*Plac8*) was upregulated 2.9-fold at P0, and is also involved in pancreatic cancer progression [41]. Interestingly, *Plac8* is highly expressed in the non-granulated cells of neonatal rat islets, whereas it is expressed at far lower levels in neonatal and adult beta cells (E-GEOD-47174). Notably, *Agr2* and *Plac8* are not temporally regulated from E20 to P2 in the control pancreas.

### 2.7. Pathway Analysis of Transcripts Regulated by Low-Protein Diet

**Figure 7:**
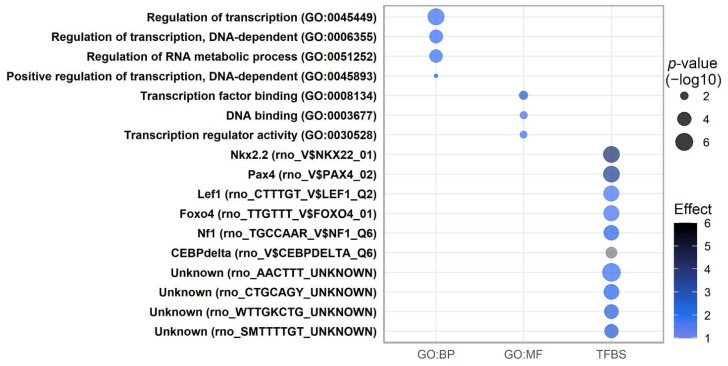
Gene Ontology biological processes (GO:BP) and molecular functions (GO:MF), which are significantly over-represented in the gene lists of downregulated genes caused by the low-protein diet, and significantly enriched transcription factor binding sites (TFBS) based on downregulated genes caused by the low-protein diet during gestation.

Lists of differentially expressed genes were divided in up- and downregulated LP genes (relative to CO) regardless of the time point. Biological process (BP) and molecular function (MF) Gene Ontology terms were tested for enrichment. The most significant MF category for genes downregulated in the LP group was transcription factor binding (*p* = 4.7 × 10^−6^) (Figure 7), while no there were no significantly enriched categories for upregulated transcripts when adjusting for multiple testing. In the BP category, the regulation of transcription (*p* = 2.0 × 10^−9^) was the most enriched for downregulated LP genes. These findings could indicate an overall reduction in transcription and a lowered capacity of LP animals to expand and maturate the pancreas. No metabolic categories were identified as enriched, which was somewhat surprising because of the difference in nutrient supply during the fetal stage and the shift from high glucose supply during gestation into high fat substrate supply from the mother’s milk in the suckling period.

A large number of TFBS motifs were highly enriched among downregulated transcripts in the LP pancreas, which was concordant with the downregulated genes being highly enriched in processes involving the regulation of transcription. Interestingly, four motifs with uncharacterized protein binding partners are among the 10 most enriched TFBS motifs (AACTTT, CTGCAGY, WTTGKCTG, and SMTTTTGT). Among the known motifs are *NK2 homeobox 2* (*Nkx2.2*), *paired box 4* (*Pax4*), *lymphoid enhancer binding factor 1* (*Lef1*), *forkhead box O4* (*Foxo4*), *forkhead box D3* (*Foxd3*), and *neurofibromin 1* (*Nf1*). Nkx2.2, Pax4, and Lef1 are known to be involved in pancreatic islet differentiation [42], whereas a specific role of Foxo4 or Foxd3 has not been described in the pancreas. The enrichment for the AACTTT motif stems in part from the presence of sites in promoters of *Etv5*, *Hnf3* (*Foxa2*), SRY box transcription factor 4 (*Sox4*), nuclear receptor subfamily 1 group D member 1 (*Nr1d1*), bone morphogenetic protein receptor type 2 (*Bmpr2*), nuclear receptor subfamily 2 group F member 2 (*Nr2f2*), *Stat3*, activin A receptor type 1C (*Acvr1c*), RAR-related orphan receptor A (*Rora*), *Notch1,* and forkhead box O1 (*Foxo1*), all of which are downregulated by LP. For transcripts upregulated by LP, only SRF binding sites (serum response factor, SRF_Q4) were over-represented in their promoters (not shown).

## 3. Discussion

The perinatal period is characterized by numerous metabolic changes and the maturation of the endocrine system. In the endocrine rat pancreas, a considerable increase in beta cells occurred between embryonic day 20 (E20) and two days after birth (P2) [10,43]. However, a comprehensive analysis of global gene expression patterns during this period had not previously been published. Thus, microarray analyses were performed at E20, immediately after birth (P0), and two days after birth (P2) to characterize the transcriptional profile of the pancreas during this period of beta cell expansion. In the normal pancreas, the major transcriptional changes occurred between E20 and P0. The Gene Ontology analysis pinpointed several lipid-related processes to be regulated prior to birth, which reflect the adaptive events preparing the animal for a change in substrate supply after birth. However, it is also possible that a downregulation of the sterol and lipid metabolic processes precedes the functional maturation of the beta cells in the perinatal rat pancreas. The transcription factors Srebf1 and Srebf2 are master regulators controlling the activity of the cholesterol- and lipid-metabolizing pathways, which are characterized by the cluster of transcripts that are markedly downregulated from E20 to P2 in the normal pancreas (Figure 3E), and both have been associated with multiple pathways required for the regulation of beta cell function. Thus, the overexpression of Srebf1 and Srebf2 has been shown to cause beta cell dysfunction and eventually diabetes, which at the cellular level is reflected by the repression of *Pdx1* expression, impaired insulin secretion and content, as well as cellular triglyceride and cholesterol accumulation [33,34]. In contrast, Srebf1 is also required for proper glucose sensing and the adult beta cell response to high glucose [44]. Notably, microRNA-21 was shown to be upregulated at birth in the rat pancreas, and Srebf1 was verified as a target for this microRNA, emphasizing the importance of the downregulation of *Srebf1* around the time of birth [45]. Since the growth rate of the exocrine compartment declines from E21 onward, while the total mass of islet tissue continues to expand at a high rate until one day after birth [46], the observed downregulation at the whole organ level of cholesterol and lipid metabolic processes is not due to an increased exocrine/endocrine ratio.

Both LP and CO groups showed dramatic increases in expression of Reg3a/3b genes from P0 to E20 (Table 1), indicating an important role in the maturation of the pancreas. Reg proteins were identified more than 40 years ago and have been implicated in several diseases, including diabetes (reviewed by [47]). So far, 4 groups of Reg proteins have been identified with considerable homology [48]. Reg proteins have been found to stimulate pancreatic beta cell growth and development, and are upregulated after gastric bypass in T2D patients [49]. The increased expression in the LP pancreas before delivery may suggest a compensatory maturation of the reduced beta cell mass.

This study also shows that *Gas6* and *Afp* become upregulated at birth in the rat pancreas. These genes are associated with the progenitor cell compartment during liver regeneration. Gas6, a vitamin-K-dependent ligand for Axl receptor tyrosine kinase, is generally considered a growth and survival factor and has been implicated in hepatic regeneration, possibly favoring hepatic progenitor cell accumulation [23]. Here, *Gas6* expression was decreased at P2 in the LP pancreas. Furthermore, Gas6 was shown to promote beta cell replication without affecting apoptosis, suggesting that the transient upregulation of *Gas6* at birth contributes to the increase in beta cell proliferation after birth [29]. Interestingly, its receptor Axl was upregulated at P2 in the LP pancreas, which may contribute to the delayed maturation of the beta cells, since an inhibitor of Axl (R428) was found to promote MAF BZIP transcription factor A (MafA) expression in human embryonic stem cell differentiation to beta cells [50]. Afp is a marker of fetal hepatoblasts and adult liver progenitor cells [51,52], which is involved in the regulation of developing cells [53] and is regulated during pancreas development [54]. Similar molecular mechanisms may exist to regulate the activation of progenitors in the liver and pancreas. At P0, Afp was localized to the endocrine compartment and appeared to be associated with vessels and intra-islet capillaries, which have been suggested to be important for the regulation of the beta cell mass and function [55,56]. This could suggest a role of Afp-expressing cells in the complex interactions between endothelial cells and beta cells, which are important for regulating both beta cell expansion and function [55,56]. It would be very relevant to further characterize the role of this protein in the endocrine pancreas with the aim of identifying targets to utilize in regenerative medicine. Afp has previously been shown to have immune-suppressive actions during fetal development [57]. In addition, the novel candidates for a role in the regulation of the functional beta cell mass include *Dusp6* (decreased by LP diet) and *Angptl4*. Like *Afp* and *Gas6*, they are markedly more expressed in neonatal endocrine progenitor cells while being downregulated in adult beta cells (E-GEOD-47174). Dusp6 is a member of the dual-specificity protein phosphatase subfamily, which specifically inactivates the MAP kinase MAPK1/ERK2, thereby regulating cellular proliferation and differentiation [16]. Angptl4 is a member of the angiopoietin/angiopoietin-like gene family and is a secreted protein, which is directly involved in regulating glucose and lipid homeostasis as well as insulin sensitivity in adipose tissue and is an anti-apoptotic factor for vascular endothelial cells [17,58].

Furthermore, Lgmn and Etv5 are novel potential candidates in the complex regulation of the beta cell mass in the perinatal pancreas. Lgmn is a lysosomal cysteine protease, which is involved in extracellular matrix remodeling by degrading fibronectin [24] and regulating the activity of matrix metalloproteinases (MMPs), especially MMP-2 [59], as well as protecting against apoptosis and facilitating tumor growth and invasiveness [25]. In addition, Lgmn has been implicated in pancreas regeneration [26]. Etv5 is a transcription factor suggested to be involved in stem or progenitor cell maintenance in the testes [27], and has been linked to pancreatic endocrine development [28]. In this study, Etv5 was decreased in the rat pancreas around the time of birth by the LP diet, and it was recently shown that Etv5-deficient mice have smaller islets and reduced insulin exocytosis [60]. Like Lgmn, Etv5 is associated with the regulation of MMP-2 activity [61]. Since MMP-2 has been implicated in proper islet morphogenesis [62,63] and suggested as a marker for newly formed beta cells [11], and in light of the roles of Lgmn and Etv5 in tumor invasiveness and metastasis [64,65], it is possible that these factors may be involved in the assembly of newly formed islets by facilitating the migration of endocrine (precursor) cells [66]. Interestingly, the trefoil factors *TFF1* and *TFF3* were markedly upregulated in the fetal rat pancreas on an LP diet around the time of birth, suggesting that they promote the maturation of the beta cells [30]. We have previously found that delta-like-1 (*Dlk1*), fetal antigen-1 (*FA-1*), and preadipocyte factor-1(*Pref-1*) were upregulated by GH and prolactin (PRL), but that Dlk1 suppressed the proliferation in neonatal islets [67]. This is in accordance with the present finding that dlk1 is downregulated in perinatal islets in the offspring of LP dams (Supplementary Table S1), suggesting a compensatory maturation of the glucose-induced insulin secretion.

The low protein availability during gestation altered a large number of the transcripts, most of which were decreased at one or more time points. The pathways affected by the downregulated transcripts were related mainly to the regulation of gene transcription (Figure 7), which is consistent with transcription factor binding as an enriched term for molecular function. These biological pathways are consistent with the theory that the LP diet causes impaired and premature islet formation. Further support for this hypothesis is the observation that the beta cell progenitor transcription factor *Neurog3* is markedly downregulated by the LP diet (Figure 4C). Furthermore, the analysis of the proximal promoter motifs shared by downregulated transcripts showed that four motifs with no recognized protein binding partner were significantly enriched. Especially the site AACTTT is interesting, which was shared by *Etv5, Hnf3, Sox4, Nr1d1, Bmpr2, Nr2f2, Stat3, Acvr1c, Rora, Notch1,* and *Foxo1*, because these transcripts either encode transcription factors involved in promoting beta cell replication or function or the proteins involved in morphogenesis and islet formation. Nkx2.2, Pax4, and Lef1 binding sites were also enriched in promoters of downregulated transcripts. Collectively, the pathways affected by LP could indicate an endocrine pancreas with a less active apparatus that is necessary for proper development and expansion via neogenesis and proliferation. A relatively low number of transcripts were upregulated by the LP diet, the most notable of which were *Agr2*, upregulated 44-fold at P2, and *Plac8,* upregulated 3-fold at P0, which are both involved in cell migration and metastasis.

In conclusion, by characterizing the global gene expression profile during the burst of beta cell formation and functional maturation in the perinatal rat pancreas, we identified novel candidate genes and pathways that may play fundamental roles in the regulation of the functional beta cell mass. This may be of relevance to the generation of functional beta cells in vitro from either embryonic stem cells (ES) or induced pluripotent stem cells (iPS). One of the major obstacles at present is to achieve a fully functional glucose sensing phenotype capable of secreting insulin in adequate amounts ex vivo that can subsequently be used for cell-based therapies [50,68]. Thus, we deposited a data set that may be valuable in the generation of fully functional beta cells from embryonic stem cells or pancreatic progenitor cells in vitro to be used in replacement therapy.

Maternal malnutrition has severe effects on the offspring. A reduced beta cell mass following in utero malnourishment, may, when exposed to increased weight gain, lead to gestational diabetes that may result in macrosomia and a risk of T2D for both the mother and child. Undernutrition during pregnancy may lead to insufficient nourishment of the offspring and reduced beta cell mass in the offspring, with an increased risk of T2D later in life. It is well known that both low and high birthweights predispose the offspring to T2D.

Some of the factors mentioned in this paper may have the therapeutic ability to prevent the loss of beta cells or stimulate the proliferation or neogenesis of beta cells. The promising candidates are *TFF3*, *Gas6*, *Trh*, and factors that stimulate *Neurog3* expression.

## 4. Materials and Methods

**Animal study.** Wistar rats, aged 10–11 weeks, were time-mated at Taconic, Denmark, and transferred to local facilities the day after mating. The animals were randomly assigned one of either two isocaloric diets: a low-protein diet (8% casein; LP; Hope Farms 4400.00, Woerden, NL) or normal-protein diet (20% casein, ‘Ctrl’, Hope Farms 4400.01). Animals were pair-fed and allowed free access to drinking water, and kept under a 12 h light–12 h dark cycle until they were killed. The animal studies were conducted in accordance with institutional guidelines and approved by the Danish Animal Experiments Inspectorate.

**Tissue samples.** The pancreata were excised at embryonic day 20 (E20), immediately after birth (P0), and two days after birth (P2). After decapitation, the individual pancreata were quickly dissected and placed directly in ice-cold TRI Reagent (Sigma-Aldrich, St Louis, MO, USA). In three separate experiments, we dissected the pancreas from one litter at days E20, P0, and P2.

**RNA extraction.** The pancreas samples from all pups were kept individually to enable equalized pooling of the RNA for the subsequent array analyses, and we pooled equal amounts of RNA from 3 male pups and 3 female pups for each array for a total of 2 × 9 arrays (Ctrl and LP conditions), with triplicate measurements at each time point. The total RNA was extracted with TRI Reagent. The RNA samples from individual pancreata were kept separate in order to verify the array results in both pools of RNA and in individual animals via real-time qPCR. The quality of each RNA sample was determined on the 2100 Bioanalyzer (Agilent Technologies, Santa Clara, CA, USA). Only samples with a 28S/18S RNA ratio >2 and RNA integrity number >7 were used. The RNA was quantified using NanoDrop (Thermo Scientific, Waltham, MA, USA).

**Microarray analysis.** Equal amounts of RNA from each of 3–4 male and 3–4 female littermates were pooled at each time point (E20, P0, and P2), and the quality of the RNA pools was determined as described above. The biotin-labeled cRNA was synthesized according to the Affymetrix protocol and hybridized to the Rat Genome 230 2.0 Array (Affymetrix, Santa Clara, CA, USA). This was repeated in each of the three biological replicate experiments.

**Data processing and statistics.** Presence calls for all array data were obtained using MAS5 and GC Robust Multi-Array Average (gcRMA) was used for normalization. We used Biometric Research Branch (BRB) ArrayTools version 3.6.0 software for the analysis of the time series of the normal pancreas tissue to filter and complete the statistical analysis [69]. Multivariate permutation tests [70] were applied to identify significantly differently regulated genes while controlling the proportion of false discoveries. The maximum proportion of false discoveries was set to 0.1 with a 90% confidence level. For the evaluation of the differential expression of transcripts in LP vs. CO groups, a significance analysis of microarrays (SAM) [71] was used (samR package using the statistical environment R and the Bioconductor module). The microarray data are available in Supplementary Table S1. Genes that were different between LP E20 vs. CO E20, LP P0 vs. CO P0 and LP P2 vs. CO P2 with a false discovery rate (FDR) of less than 10% were chosen for further analysis. The resulting list of significantly expressed genes were then imported into dChip [72] and used for unsupervised hierarchical clustering. Settings: Distance measure of one minus the Pearson correlation coefficient and average linkage with a *p*-value threshold of 0.01 for significant sample clusters and a *p*-value threshold of 0.001 for significant gene clusters.

**Promoter analysis.** The analysis of over-represented functional categories was performed with Functional Annotation Clustering using Database for Annotation Visualization and Integrated Discovery (DAVID) [73,74]. The identification of over-represented promoter cis-elements in the promoters of differentially regulated genes was performed in R using pcaGOPromoter [75]. Promoter sequences from −1350 to +150 were extracted using the UCSC Table browser http://genome.ucsc.edu/and the over-representation of promoters with hits for a given matrix was calculated using Fishers exact test for proportions. The position weight matrices were annotated to transcription factor binding sites stored in the JASPAR database (http://JASPAR.genereg.net/) [76] and TRANSFAC database [77]. For the analysis of RTqPCR data, an ANOVA or two-way ANOVA was used as appropriate, with post hoc individual comparisons using either Tukey or Bonferroni corrections.

**Quantitative real-time PCR.** The RTqPCR was performed on 10× diluted cDNA reverse transcribed from 1 µg total RNA with qScript™ cDNA SuperMix (Quanta, Gaithersburg, MD, USA) on a LightCycler 2.0 instrument (Roche, Basel, Switzerland) using LightCycler^®^ FastStart DNA MasterPLUS SYBR Green I. The gene expression was normalized to ribosomal protein L13A (*Rpl13a*), which had a constant expression level over the three time points examined. The oligonucleotide sequences are listed in Supplementary Table S2. GraphPad Prism was used for the statistical analysis and *p* < 0.05 was considered significant.

**In situ hybridization (ISH).** ISH was performed on sections from paraffin-embedded perinatal rat pancreas samples with a digoxigenin-labeled LNA™ probe targeted against the same rat Afp sequence as the primers for the qPCR (Exiqon, Vedbaek, Denmark). Briefly, the slides were passed through graded ethanol to water, rinsed in DEPC/PBS (44 °C, 2 × 5 min), pre-treated with proteinase K (15 min), and immersed in 0.2% glycine for 30 s. The slides were then washed thoroughly in DEPC/PBS (44 °C) and post-fixed in 4% PFA for 10 min followed by washing in DEPC/PBS for 4 × 2 min and for 2 × 5 min in 0.25% acetic anhydride in 0.1 M triethanolamine. Subsequently, the slides were rinsed in DEPC/PBS for 4 × 2 min and pre-hybridized for 2 h in hybridization mixture at 52 °C, then incubated overnight with the Afp probe at 52 °C. The control sections were treated with 1 mg/mL RNase A (Sigma-Aldrich, St Louis, MO, USA) in PBS for 1 h at 37 °C prior to hybridization. The slides were then washed in 0.1 × SSC at 62 °C for 1 × 10 min and for 2 × 10 min at 56 °C, followed by washing in 2 × SSC for 5 min and quenching in 3% H2O2 for 10 min at RT, with subsequent washing in TN buffer for 3 × 3 min at pH 7.5. Non-specific binding was blocked with blocking buffer (CAS-Block from Spot-Light CISH Polymer detection kit, Invitrogen, San Diego, CA, USA) and the immunohistochemical detection of digoxigenin was achieved with Zymed (Invitrogen, San Diego, CA, USA). The negative controls included sections without a probe and sections with a scrambled control probe (Exiqon, Vedbaek, Denmark).

**Immunohistochemistry (IHC).** The tissue used for IHC was fixed in 4% formaldehyde overnight (o/n) followed by embedding in paraffin. The sections (3 μm) were blocked with Protein Block (Dako, Dako, Glostrup, Denmark), incubated with the primary antibody against alpha-fetoprotein o/n (Afp, monoclonal mouse anti human, 1:200, Abcam, Cambridge, UK) or Neurog3 (poly-clonal rabbit antibody, 1:200, kindly provided by Professor Michael German, M.D; Department of Medicine, UCSF Diabetes Center, CA, USA) and secondary antibodies for 2 h. The secondary antibody was labeled with horseradish peroxidase (HRP) and developed by AEC (9-Ethylcarbazol-3-amine). The nuclei were labeled with hematoxylin. The antigen retrieval was performed with proteinase K treatment (10 mg/mL, Sigma-Aldrich, St Louis, MO, USA). The controls for specificity included sequential dilutions of the primary antibody and omission of the primary antibody.

## Figures and Tables

**Table 1:** Fold changes (FCs) in most up- or downregulated annotated transcripts in control (CO) diet perinatal pancreas samples with corresponding changes listed for low-protein (LP) diet samples.

	Increased at P0 vs. E20				Increased at P2 vs. P0		
Gene Symbol	Gene Name	FC CO	FC LP	Gene Symbol	Gene Name	FC CO	FC LP
*Reg3a/3b*	Regenerating family member 3 alpha/beta	461/335	617/617	*LOC688750*	CD209 antigen	5.1	1.8
*Hmgcs2*	3-hydroxy-3-methylglutaryl-Coenzyme A synthase 2	270	55	*Prss35*	Protease, serine, 35	4.9	1.2
*LOC286960*	Preprotrypsinogen IV	155	103	*C5*	Complement C5	4.8	−1.3
*LOC312273*	Trypsin V-A	145	151	*LOC500183*	NGF-binding Ig light chain	4.7	1.3
*Gif*	Gastric intrinsic factor	61	61	*C4a*	Complement component 4a	3.6	1.6
*Alb*	Albumin	58	12	*LOC365985*	Adenylate kinase 5 isoform 1	3.4	1.6
*Angptl4*	Angiopoietin-like 4	54	18	*Tinag*	Tubulointerstitial nephritis antigen	3.1	−1.5
*Apoa1*	Apolipoprotein A1	39	117	*Spink3*	Serine protease inhibitor, Kazal type 3	2.9	1.7
*Try10*	Pancreatic trypsin 1	30	15	*LOC686268*	SUMO/sentrin specific protease 5	2.9	1.4
*Ahsg*	Alpha-2-HS-glycoprotein	29	19	*Cuzd1*	CUB and zona pellucida-like domains 1	2.9	1.1
*Spink1*	Serine protease inhibitor, Kazal type 1	29	11	*Egfl6*	EGF-like-domain, multiple 6	2.9	2.0
*Kng1*	Kininogen 1	28	23	*Ak7*	Adenylate kinase 7	2.8	−1.1
*Gas6*	Growth arrest specific 6	28	18	*Nradd*	Neurotrophin receptor associated death domain	2.8	1.3
*Apob*	Apolipoprotein B	25	84	*Ret*	Ret proto-oncogene	2.7	−1.0
*Slc18a2*	Solute carrier family 18 (vesicular monoamine), member 2	23	5.3	*Zcchc12*	Zinc finger, CCHC domain containing 12	2.6	−1.1
	**Decreased at P0 vs. E20**			**Decreased at P2 vs. P0**			
**Gene Symbol**	**Gene Name**	**FC CO**	**FC LP**	**Gene Symbol**	**Gene Name**	**FC CO**	**FC LP**
*Serpina6*	Serpin family A member 6	−47	−74	*Ahsg*	Alpha-2-HS-glycoprotein	−31	−1.4
*Hbe1*	Hemoglobin subunit epsilon 1	−44	−30	*Fga*	Fibrinogen, alpha polypeptide	−23	−1.2
*Tinag*	Tubulointerstitial nephritis antigen	−30	−7.6	*Fabp1*	Fatty acid binding protein 1	−23	−1.3
*Tnni*	Troponin I, skeletal, slow	−28	−23	*Kng1*	Kininogen 1	−21	−1.6
*Hbg1*	Hemoglobin, gamma A	−27	−19	*Apoc2*	Apolipoprotein C-II	−19	−4.6
*Hdc*	Histidine decarboxylase	−24	−17	*Fgg*	Fibrinogen, gamma polypeptide	−17	2.0
*Ptges*	Prostaglandin E synthase	−22	−14	*Apoc1*	Apolipoprotein C-I	−16	−1.2
*Tm7sf2*	Transmembrane 7 superfamily member 2	−17	−4.1	*Apoh*	Apolipoprotein H	−15	−1.0
*Pln*	Phospholamban	−17	−4.2	*Fgb*	Fibrinogen, beta polypeptide	−13	−1.1
*Nags*	N-acetylglutamate synthase	−17	−3.2	*Apoa1*	Apolipoprotein A-I	−13	−5.4
*Clic3*	Chloride intracellular channel 3	−13	−7.1	*Hpx*	Hemopexin	−13	−1.2
*Adam2*	A disintegrin and metalloprotease domain 2	−12	−9.8	*LOC299282*	Serine protease inhibitor	−12	−1.3
*Fdft1*	Farnesyl diphosphate farnesyl transferase 1	−12	−4.4	*Itih3*	Inter-alpha trypsin inhibitor, heavy chain 3	−10	−1.2
*LOC682690*	Chromodomain helicase DNA binding protein 9	−11	1.2	*Pck1*	Phosphoenolpyruvate carboxykinase 1	−9.9	−5.3
*Camkk2*	Calcium/calmodulin-dependent protein kinase kinase 2, beta	−11	−2.4	*Serpina3k*	Serine peptidase inhibitor, clade A, member 3K	−9.7	−1.2

Relative transcript abundances at the respective time points, sorted by differential expression in the CO diet. The top 15 up- or downregulated transcripts at each time point comparison are listed. Downregulation is denoted as a negative fold change. Only robustly expressed transcripts (intensity above 100 in at least one time point) were included.

**Table 2:** Differential expression caused by the low-protein (LP) diet at E20, P0, and P2 in the 10 most up- or downregulated transcripts.

Gene Symbol	Gene Name	E20	Gene Symbol	Gene Name	P0	Gene Symbol	Gene Name	P2
Increased	LP vs. Control (Fold Regulation)		Increased	LP vs. Control (Fold Regulation)		Increased	LP vs. Control (Fold Regulation)	
*Agtr2*	Angiotensin II receptor, type 2	11	*Apoa4*	Apolipoprotein A4	142	*Lgals4*	Lectin, galactose binding, soluble 4	236
*LOC686892*	Muscleblind-like 1 isoform d	8.7	*Pga5*	Pepsinogen 5	112	*Pga5*	Pepsinogen 5	215
*Cav*	Caveolin	8.6	*Rbp2*	Retinol binding protein 2	86	*Agr2*	Anterior gradient 2	110
*Ppp3r1*	Calcineurin B, type I	8.5	*Lgals4*	Lectin, galactose binding, soluble 4	79	*Retnla*	Resistin like alpha	93
*Zfp260*	Zinc finger protein 260	7.7	*Tff1*	Trefoil factor 1	73	*Fabp1*	Fatty acid binding protein 1	92
*Sept2*	Septin 2	7.6	*Clca3*	Chloride channel calcium activated 3	55	*Tff1*	Trefoil factor 1	87
*Ogn*	Osteoglycin	7.4	*Agr2*	Anterior gradient 2	48	*Gkn1*	Gastrokine 1	82
*Mat2a*	Methionine adenosyltransferase II, alpha	6.3	*Fabp2*	Fatty acid binding protein 2	46	*LOC56825*	Prochymosin	66
*Il13ra1*	Interleukin 13 receptor, alpha 1	5.9	*Clca6*	Chloride channel calcium activated 6	45	*Clca3*	Chloride channel calcium activated 3	42
*LOC498358*	Solute carrier family 30 (zinc transporte), member 9	5.6	*Gkn1*	Gastrokine 1	42	*Sult1b1*	Sulfotransferase family 1B	39
**Decreased**	**LP vs. Control (Fold Regulation)**	**E20**	**Decreased**	**LP vs. Control (Fold Regulation)**	**P0**	**Decreased**	**LP vs. Control (Fold Regulation)**	**P2**
*Fos*	FBJ murine osteosarcoma viral oncogene	−14	*Myo5c*	Myosin Vc	−5.1	*Atp8b1*	ATPase, Class I, type 8B, member 1	−11
*LOC680231*	Chromodomain helicase DNA binding protein 9	−12	*Phlda1*	Chromodomain helicase DNA binding protein 9	−4.3	*Aff4*	AF4/FMR2 family, member 4	−9.9
*Zfhx1b*	Zinc finger homeobox 1b (ZEB2)	−6.8	*Gtl2*	GTL2, imprinted maternally expressed untranslated	−4.2	*Foxo1a*	Forkhead box O1A	−9.6
*Atxn2*	Ataxin 2	−6.7	*Smoc1*	SPARC-related modular calcium binding protein 1	−4.1	*Eif2c2*	Eukaryotic translation initiation factor 2C, 2	−9.4
*RGD1561386*	CBL E3 ubiquitin protein ligase	−6.4	*LOC682488*	Ras-related protein Rab-1B	−4.1	*Tns*	Tensin	−9.0
*Adipor2*	Adiponectin receptor 2	−6.1	*Lamc1*	Laminin, gamma 1	−3.9	*Akap9*	A kinase (PRKA) anchor protein 9	−8.9
*Eif2c2*	Eukaryotic translation initiation factor 2C, 2	−5.8	*Adhfe1*	Alcohol dehydrogenase, iron containing, 1	−3.9	*Ash1l*	Absent, small, or homeotic)-like	−8.8
*Mt1a*	Metallothionein 1a	−5.8	*Fgfr1*	Fibroblast growth factor receptor 1	−3.8	*Rck*	DEAD box protein *rck*/p54	−7.4
*Tbl1x*	Transducin (beta)-like 1 X-linked	−5.8	*P34*	P34 protein	−3.7	*Tbl1x*	Transducin (beta)-like 1 X-linked	−7.4
*Atp8b1*	ATPase, Class I, type 8B, member 1	−5.4	*Ccnl2*	Cyclin L2	−3.5	*Ubn1*	Ubinuclein 1	−6.7

Relative transcript abundances at the respective time points. Downregulation is denoted as a negative fold change. Only robustly expressed transcripts (intensity above 100 for at least one time point) were included.

## Data Availability

All microarray data in processed form are available in Supplementary Table S1.

## Supplementary Materials

The supporting information can be downloaded at: https://www.mdpi.com/article/10.3390/ijms231911057/s1.

## Abbreviations

bHLH, basic helix loop helix; E20, embryonic day 20; GO, Gene Ontology; LP, low-protein; CO, control; P0, day of birth; P2, two days postnatal; PWM, position weight matrix; TF, transcription factor; TFBS, transcription factor binding site.
