# Supplementary material for: Fetal Programming of the Endocrine Pancreas: Impact of a Maternal Low-Protein Diet on Gene Expression in the Perinatal Rat Pancreas
Source: Int J Mol Sci. 2022 Sep 21;23(19):11057. doi: 10.3390/ijms231911057 (PMC9569808; doi:10.3390/ijms231911057)
Supplement: Supplementary file 1 [file ijms-23-11057-s001.zip › Supplementary Table S2 Oligonucleotide sequences.docx]

Table 1 Oligonucleotide sequences used for primers

| Transcript | Forward primer | Reverse primer |
| --- | --- | --- |
| Ribosomal protein L13A (Rpl13a) | 5’-ccaccctatgacaagaaaaagc-3’ | 5’-acattcttttctgcctgtttcc-3’ |
| Insulin2 (Ins2) | 5’-tgcccaggcttttgtca-3’ | 5’-ctccagttgtgccacttgt-3’ |
| Neurogenin3 (Ngn3) | 5’-agggatacctgaccacttgcta-3’ | 5’-cagttctgagccagtcacaaag-3’ |
| Pancreatic and duodenal homeobox 1 (Pdx1) | 5’-aaccggaggagaataagaggac-3’ | 5’-ttgtcccgctactacgtttctt-3’ |
| Pancreatic lipase (Pnlip) | 5’-cctaatggagggatggaaatg-3’ | 5’-gctacaggagaagccagagaag-3’ |
| Branched chain amino-acid transaminase 2 (Bcat2) | 5’-cctcctgttcgtcattctgtg-3’ | 5’-gcctttcttctgggcttctt-3’ |
| Alpha-fetoprotein (Afp) | 5’-ttagactcttcccaatgcc-3’ | 5’-gctatctgtgtttctggatg-3’ |
| Sterol regulatory element binding protein 1 (Srebf1) | 5’-cccctgggcctggaatcaaaga-3’ | 5’-gggtgttcccaggaagggtt-3’ |
| Sterol regulatory element binding protein 2 (Srebf2) | 5’-ggagtcaggttctggaggctgg-3’ | 5’-cgcccggcccaaaacggata-3’ |
| Early growth response factor 1 (Egr1) | 5’-ctttcctcactcacccacca-3’ | 5’-cgttattcagagcgatgtcaga-3’ |
|  |  |  |
